# Five-Year-Olds’ Systematic Errors in Second-Order False Belief Tasks Are Due to First-Order Theory of Mind Strategy Selection: A Computational Modeling Study

**DOI:** 10.3389/fpsyg.2017.00275

**Published:** 2017-02-28

**Authors:** Burcu Arslan, Niels A. Taatgen, Rineke Verbrugge

**Affiliations:** Institute of Artificial Intelligence, University of GroningenGroningen, Netherlands

**Keywords:** second-order false belief reasoning, theory of mind, instance-based learning, reinforcement learning, computational cognitive modeling, ACT-R

## Abstract

The focus of studies on second-order false belief reasoning generally was on investigating the roles of executive functions and language with correlational studies. Different from those studies, we focus on the question how 5-year-olds select and revise reasoning strategies in second-order false belief tasks by constructing two computational cognitive models of this process: an instance-based learning model and a reinforcement learning model. Unlike the reinforcement learning model, the instance-based learning model predicted that children who fail second-order false belief tasks would give answers based on first-order theory of mind (ToM) reasoning as opposed to zero-order reasoning. This prediction was confirmed with an empirical study that we conducted with 72 5- to 6-year-old children. The results showed that 17% of the answers were correct and 83% of the answers were wrong. In line with our prediction, 65% of the wrong answers were based on a first-order ToM strategy, while only 29% of them were based on a zero-order strategy (the remaining 6% of subjects did not provide any answer). Based on our instance-based learning model, we propose that when children get feedback “Wrong,” they explicitly revise their strategy to a higher level instead of implicitly selecting one of the available ToM strategies. Moreover, we predict that children’s failures are due to lack of experience and that with exposure to second-order false belief reasoning, children can revise their wrong first-order reasoning strategy to a correct second-order reasoning strategy.

## Introduction

The ability to understand that other people have mental states, such as desires, beliefs, knowledge and intentions, which can be different from one’s own, is called theory of mind (ToM; Premack and Woodruff, 1978). Many studies have shown that children who are younger than four have problems to pass verbal tasks in which they are expected to predict or explain another agent’s behavior in terms of the agent’s mental states, such as false beliefs (Wellman et al., 2001; see Onishi and Baillargeon, 2005 for an example of a non-verbal false belief task). In our daily lives, we do not only take the perspective of another agent (first-order ToM) but also use this ToM recursively by taking the perspective of an agent who is taking the perspective of another agent. For example, if David says, “Mary (falsely) *believes that* John *knows that* the chocolate is in the drawer,” he is applying second-order ToM by attributing a mental state to Mary who is attributing another mental state to John. While children start to pass verbal first-order ToM tasks around the age of four, it takes them a further one to 3 years to pass second-order ToM tasks (Perner and Wimmer, 1985; Sullivan et al., 1994; for a review, see Miller, 2009, 2012). Why can children not pass second-order ToM tasks once they are able to pass first-order ToM tasks? The central focus of this study is to provide a procedural account by constructing computational cognitive models^1^ to answer this question.

Many studies have shown that children who are younger than four are make systematic errors in verbal first-order false belief tasks (Wellman et al., 2001). A prototype of verbal first-order false belief task is as follows: “Ayla and Murat are sister and brother. They are playing in their room. Their mother comes and gives chocolate to Murat but not to Ayla, because she has been naughty. Murat eats some of his chocolate and puts the remainder into the drawer. He doesn’t give any chocolate to Ayla. She is upset that she doesn’t get any chocolate. After that, Murat leaves the room to help his mother. Ayla is alone in the room. Because she is upset, she decides to change the location of the chocolate. She takes the chocolate from the drawer, and puts it into the toy box. Subsequently, Murat comes to the room and says he wants to eat his chocolate.” At this point, the experimenter asks a first-order false belief question: “Where will Murat look for his chocolate?” Children who are able to give the correct answer by saying “in the drawer,” correctly attribute a false belief to Murat, because he does not know that Ayla put the chocolate into the toy box. If children do not know the answer to the first-order false belief question and simply try to guess the answer, they can randomly report one of the two locations: “drawer” or “toy box.” Interestingly, most 3-year-old children do not give random answers but make systematic errors by reporting the real location of the chocolate (zero-order ToM) instead of reporting the other character’s false belief (first-order ToM). This systematic error is generally called ‘reality bias’^2^ (Mitchell et al., 1996).

There are two dominant explanations in the first-order ToM literature for 3-year-olds’ ‘reality bias.’ The first explanation proposes that children do not distinguish the concept of beliefs from reality, thus children need a *conceptual change* (Wellman and Gopnik, 1994; Wellman et al., 2001). The second explanation proposes that children’s systematic error is due to the fact that reality is more salient to them, thus children’s failure in verbal tasks are in general due to the *complexity* of the tasks, which adds further processing demands on children’s reasoning processes (Carlson and Moses, 2001; Hughes, 2002; Birch and Bloom, 2004, 2007; Epley et al., 2004). More specifically, children automatically reason about their own perspective and in order to give an answer about another agent’s perspective which is different from the reality, they should first inhibit their own perspective and then take into account the other agent’s perspective and give an answer accordingly (Leslie and Polizzi, 1998; Leslie et al., 2004, 2005). The debate is still on about the possible reasons of children’s ‘reality bias’ (Hansen, 2010; Lewis et al., 2012; Helming et al., 2014; Baillargeon et al., 2015; Rubio-Fernández, 2017). In any case, it is known that most of the typically developing children around the age of 5 are able to pass first-order false belief tasks. Therefore, we can safely assume that 5-year-old children’s conceptual development of reasoning about another agent’s false beliefs and their executive functioning abilities to inhibit their own perspective are already well developed. This means that 5-year-olds have both efficient zero-order and first-order ToM strategies in their repertoire. Furthermore, we argue that although 5-year-olds are able to attribute second-order mental states to other agents, they are not used to answering questions that require second-order false belief attribution, which is why they need sufficient exposure to second-order false belief stories to revise their strategy.

**FIGURE 1:**
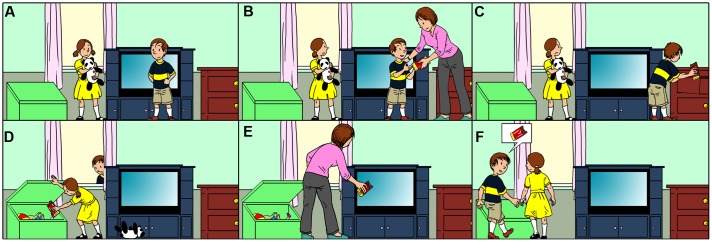
**A modified version of the standard ‘unexpected location’ second-order false belief stories (Illustration Avik Kumar Maitra)** **(A)** The first image in the first row, **(B)** The second image in the first row, **(C)** The third image in the first row, **(D)** The first image in the second row, **(E)** The second image in the second row, **(F)** The third image in the second row.

Similar to the first-order false belief tasks, second-order false belief tasks are used to assess the continuation of children’s ToM development after the age of 4. Regardless of the variations in the second-order false belief tasks (see Perner and Wimmer, 1985; Sullivan et al., 1994), they provide two critical pieces of information in addition to the first-order false belief task for which we introduced a prototype above. The first addition for the prototype story is: “While Ayla is changing the location of the chocolate, Murat passes by the window, and he sees how Ayla takes the chocolate from the drawer and puts it into the toy box.” The second additional aspect is: “Ayla does not notice that Murat sees her hiding the chocolate” (**Figure 1D**). Therefore, Ayla has a false belief about Murat’s belief about the location of the chocolate (i.e., Ayla *thinks that* Murat *believes that* the chocolate is in the drawer). The second-order false belief question for this prototype is as follows: “Where does Ayla think that Murat will look for the chocolate?”^3^. If children correctly attribute a false belief to Ayla, who *thinks* that Murat *believes* that the chocolate is in the drawer, they give the correct answer “drawer.” Otherwise, they give the wrong answer “toy box.” However, the answer “toy box” would be the correct answer to both the question “Where is the chocolate now?” (zero-order ToM), and the question “Where will Murat look for the chocolate?” (first-order ToM). That is why it is not possible to distinguish whether the wrong answer “toy box” to the second-order false belief question is due to applying a zero-order or a first-order ToM strategy.

To the best of our knowledge, there is no study that has a specific prediction together with a possible explanation about the level of ToM reasoning in children’s wrong answers in second-order false belief tasks. However, a modified version of the standard second-order false belief task in which it is possible to distinguish children’s level of ToM reasoning has been constructed (Hollebrandse et al., 2008). Following our prototype of the standard second-order false belief story that we mentioned above, a prototype of the modified version of the second-order false belief story has the following additional information: After telling the children that Ayla does not know that Murat saw her hiding the chocolate in the toy box, the children are informed that the mother of Ayla and Murat comes to the room when both Ayla and Murat are not there. The mother finds the chocolate in the toy box while she is cleaning the room, takes it out of the toy box, and puts it into the TV stand (**Figure 1E**). This modification allows us to distinguish children’s zero-order ToM answers (“TV stand”) from their first-order ToM answers (“toy box”) for the second-order false belief question “Where does Ayla think that Murat will look for the chocolate?”.

Considering our central question why children cannot pass second-order false belief tasks once they are able to pass first-order false belief tasks, a new question about strategy selection arises: Once 5-year-old children already have zero-order and first-order ToM strategies in their repertoire, do they predominantly use a zero-order ToM strategy or a first-order ToM strategy when they fail in second-order false belief tasks? There are two contradictory findings about children’s systematic errors on second-order false belief tasks. Hollebrandse et al. (2008) tested 35 American-English 7-year-old children (range: 6.1 – 7.10, mean = 6.11) with a modified version of a second-order false belief task. The goal of their study was to investigate the acquisition of recursive embedding and its possible relation with recursive ToM. Their results about the second-order false belief task showed that while 58% of the answers were based on second-order ToM strategy, 32% of the answers were based on a first-order ToM strategy, and none of the answers was based on a zero-order ToM strategy. In contrast, de Villiers et al.’s (2014) preliminary results showed that 60% of 5- to 6-year-olds’ answers were based on the zero-order ToM strategy, and only around 20% of children’s answers were based on the first-order ToM strategy in the second-order false belief task. Different from those studies, our empirical study was designed to investigate children’s level of wrong answers, and we had a model-based prediction about children’s systematic errors in second-order false belief tasks before conducting the empirical study.

Another important question is: What do children need for revising their wrong strategy to a correct second-order ToM strategy? Analogous to the first-order ToM literature, two possible explanations have been proposed for children’s development of second-order ToM: (i) *conceptual change*, and (ii) *complexity* (Miller, 2009, p. 751; Miller, 2012). The pure *conceptual change* explanation suggests that children’s failure in the second-order ToM tasks is due to their lack of realization that mental states such as beliefs can be used recursively (e.g., “John *thinks* that David *believes* that…”). On the other hand, the pure *complexity explanation* suggests that it is the higher complexity of second-order ToM reasoning that adds further demands on working memory, as does the linguistic complexity of the stories and the questions, in comparison to first-order ToM tasks.

In order to provide a procedural account for children’s ToM strategy revision, we constructed two computational cognitive models by implementing two possible learning mechanisms. The first is based on reinforcement learning (Sutton and Barto, 1998; Van Rijn et al., 2003; Shah, 2012). This type of learning is based on the utilities of the rules that carry out the possible strategies. Based on feedback, a reward/punishment is propagated back in time through the rules that have been used to make the decision. This reward/punishment mechanism updates the utility of those rules and finally the model learns to apply a correct strategy.

The second model is based on instanced-based learning (Logan, 1988; Gonzalez and Lebiere, 2005; Stevens et al., 2016). The central idea in instance-based learning is that decisions are based on past experiences that are stored in memory. Whenever a decision has to be made, the most active experience is retrieved from memory and used as the basis for the decision. Activation is based on history (how frequent and recent was the experience) and on similarity (how similar is the context of the past decision to the present experience). An advantage of instance-based learning is that feedback can be used to create an instance that incorporates the correct solution.

We used instance-based learning for the selection of different levels of ToM strategies (i.e., zero-order, first-order, second-order) that are stored in the declarative memory. When the model is correct in using a particular level of ToM, it will strengthen the instance related to that level, but when the model makes a mistake, it will add an instance for the next level.

Instead of adopting either the pure *conceptual change* or the pure *complexity* explanation, we argue that the following steps are followed. First, children should be aware that they can use their first-order ToM strategy recursively. Importantly, different from the reinforcement learning model, the instance-based learning model explicitly revises its strategy, therefore, it satisfies this condition. After that, children have to have efficient cognitive skills to carry out second-order ToM reasoning without mistakes. In the scope of this study, we assume that 5-year-olds have efficient cognitive skills to carry out the second-order ToM strategy. Finally, children need enough experience to determine that the second-order ToM strategy is the correct strategy to pass second-order false belief tasks (see Goodman et al., 2006 for a model of children’s development of first-order false belief reasoning based on experience; and Gopnik and Wellman, 1992 for the theory that children are rational agents and that with additional evidence they revise their theories, just like scientists do).

Both the reinforcement learning model and the instance-based learning model strengthen or revise their strategies based on experience and the feedback “Correct/Wrong” without further explanation. Is it possible to assume that children get feedback “Correct/Wrong” in ToM-related tasks in their everyday life? There can be many social situations in which children get the feedback “Correct/Wrong,” not from a person who gives feedback verbally, but from other consequences of a particular ToM strategy. For example, young children who are not able to apply a first-order ToM strategy are generally unable to hide themselves properly when they are playing hide and seek^4^ (e.g., they hide themselves behind the curtain while their feet are visible or basically they close their eyes with their hands without hiding themselves). In this case, the feedback “Wrong” would be conferred by the fact that the seeker finds the hider immediately. Similarly, at a later stage of development, imagine a child secretly eating some of the chocolates that his mother explicitly told him not to eat. As soon as his mother comes back to the room, the child says that he does not like chocolate with nuts. His mother gets angry and tells him to go to his room and not to join them for dinner. In this case, the child was unable to use a second-order ToM strategy (i.e., my mother *should not know that* I *know* that there are chocolates with nuts) and although he does not get any explanations, he does get the feedback “Wrong.” Note that in this example, the child also requires other types of reasoning, such as causal reasoning, in addition to second-order ToM.

The main differences between the reinforcement learning model and the instance-based learning model derive from the way they handle the feedback when the given answer is wrong. While the reinforcement learning model punishes the strategies that lead to a wrong answer, the instance-based learning model adds an instance of another strategy. This is because while the strategy selection is implicit in the reinforcement learning model, it is explicit in the instance-based learning model. Moreover, if feedback with further explanations is provided, the instance-based learning model will more likely use a second-order ToM strategy, because it explicitly increments the level of ToM strategy to a higher ToM strategy. On the other hand, the reinforcement learning model can do nothing with the further explanations. We provide more detailed explanations for these two learning mechanisms in our models in Section “The Relevant Mechanisms of the Cognitive Architecture ACT-R.” Importantly, these two models provide different predictions about children’s wrong answers in second-order false belief tasks. We present those predictions in Section “Comparing the predictions of the two models” and test them in an empirical study in Section “Experimental Validation of the Instance-Based Learning Model.”

In the following section, we first review the previous computational models of verbal first-order and second-order false belief reasoning. After that, we discuss the relevant mechanisms of the cognitive architecture ACT-R. Subsequently, we explain our instance-based and reinforcement learning models and their results and predictions.

## A Model of Second-Order False Belief Reasoning

Along with studying children’s development empirically, the modeling approach is a powerful method to provide insight into the underlying processes of children’s performance (see Taatgen and Anderson, 2002 for an example on how children learn irregular English verbs without feedback; Van Rij et al., 2010 for an example of the underlying processes of children’s poor performance on pronoun interpretation). In particular, using cognitive architectures (e.g., ACT-R: Anderson, 2007; SOAR: Laird, 2012; SIGMA: Rosenbloom, 2013; PRIMs: Taatgen, 2013) gives us the opportunity to make specific predictions about children’s accuracy, reaction times, and even the brain regions that are activated when they perform a task. These predictions can then be tested empirically.

In general, cognitive architectures have certain general assumptions about human cognition and have some parameters that are set to a default value based on previous psychological experiments to simulate average human performance. For example, it takes 200 ms to press a button on the keyboard once a decision has been made and the finger is ready to press it. In addition to these general assumptions, modelers make their own specific assumptions about the tasks that they are modeling and those assumptions can be tested empirically together with the model’s simulation results. Because it is always possible to make a fit to data by changing the parameters, it is preferable not to change these default values of the architecture and not to introduce new parameters unless there is a good explanation for doing so.

In this study, we use the cognitive architecture ACT-R (Anderson and Lebiere, 1998; Anderson, 2007). Before providing information about ACT-R and our models, in the following subsection, we review the previous computational cognitive models of verbal first-order and second-order false belief reasoning.

## Previous Models of False Belief Reasoning

Only few computational cognitive models of verbal false belief reasoning have been constructed in the literature, aiming to contribute to theoretical discussions by providing explanations. Most of those models aimed to explain children’s development of first-order false belief reasoning.

Goodman et al. (2006) approach the development of first-order false belief reasoning as rational use and revision of intuitive theories, instead of focusing on children’s limitations in processing information. By using Bayesian analysis, they simulate the transition from a model that represents children’s reasoning from their own perspective (zero-order ToM) to another model that takes into account another agent’s perspectives (first-order ToM). Initially, the zero-order ToM model is preferred due to the Bayesian Occam’s razor effect. Subsequently, based on experience with first-order false belief reasoning, the first-order ToM model becomes the preferred model thanks to its explanatory power. Bello and Cassimatis’ (2006) rule-based model showed that explicit reasoning about beliefs of another agent might not be necessary in order to pass first-order false belief tasks and that it is enough to relate people to alternate states of affairs and to objects in the world. Hiatt and Trafton (2010) simulated the gradual developmental of first-order ToM by using reinforcement learning. Their models have a good match to the available gradual development data in the literature. However, they introduced additional parameters to the core cognitive architecture, namely a “selection parameter” representing increasing functionality of the brain in children’s development, and a “simulation parameter” that determines the availability of rules for simulation in predicting another person’s action (i.e., if the simulation parameter is 0, the model is not able to predict another’s action). Therefore, the transition from zero-order ToM reasoning to first-order ToM reasoning is achieved by manipulating those parameters. More recently, Arslan et al.’s (2015b) model predicted that training children with working memory tasks might also contribute to the transition from failure to success in first-order false belief tasks.

To the best of our knowledge, there are only two computational cognitive modeling studies of second-order false belief reasoning. Wahl and Spada (2000) modeled a competent child’s reasoning steps in a second-order false belief task by using a logic programming language. Their simulations predicted that explanation of a second-order false belief attribution is more complex than its prediction. They validated their model-based prediction with an empirical study with children between the ages 6–10. For future research, they suggested to use a cognitive architecture such as ACT-R to simulate children’s incorrect answers. Recently, similar to their first-order false belief reasoning model, Hiatt and Trafton (2015) simulated the gradual developmental of second-order ToM by using reinforcement learning. Again, their model had a good match to the available data for the developmental trajectory of second-order ToM. However, they kept the “selection parameter” and “simulation parameter” that they introduced to the default parameters of ACT-R and they did not provide any specific predictions that can be tested empirically.

Different from the available second-order ToM models, we set the following criteria when constructing our models:

(i) The models should simulate children’s transitions from incorrect to correct answers in second-order false belief tasks;
(ii) The transition to second-order reasoning should naturally emerge from the simulation, and should not be controlled by mechanisms that are not part of the cognitive architecture (i.e., ACT-R);
(iii) The models should provide predictions that can be tested empirically, before conducting a behavioral experiment.

Considering the above-mentioned criteria, we explore two different learning mechanisms of ACT-R, namely instance-based learning and reinforcement learning, to be able to compare their predictions.

### The Relevant Mechanisms of the Cognitive Architecture ACT-R

ACT-R is a hybrid symbolic/sub-symbolic production-based cognitive architecture (see Anderson, 2007 for a detailed overview). Knowledge is represented in two different memory systems in ACT-R.

While the declarative memory represents the factual knowledge in the form of chunks (i.e., “The capital of France is Paris”), procedural knowledge (i.e., how to ride a bicycle) is represented by the production rules in the form of IF-THEN rules. The procedural knowledge and the factual knowledge interact when production rules retrieve a chunk from the declarative memory. At any time, the central pattern matcher checks the IF part of the production rules that match the current goal of the model, and if multiple production rules match the current goal, the rule that has the highest utility value is executed. The utility value is calculated from estimates of the cost and probability of reaching the goal if that production rule is chosen. Noise is also added to the expected utility of a production rule, making production rule selection stochastic. When a production rule is successfully executed, the central pattern matcher checks again for production rules that match the current goal. Thus, cognition unfolds as a succession of production rule executions.

For models of learning in decision making, there are two categories of solutions in ACT-R: (i) instance-based learning, (ii) reinforcement learning. *Instance-based learning* occurs by adding new chunks to the declarative memory. If an identical chunk is already in memory, the new chunk is merged with the previous identical chunk and their activation values are combined. Each chunk is associated with an activation value that represents the usefulness of that chunk. The activation value of a chunk depends on its base-level activation (*B*) and on activation sources originating in the model’s context. The base-level activation is determined by the frequency and recency of a chunk’s use together with a noise value (Anderson and Schooler, 2000). A chunk will be retrieved if its activation value is higher than a retrieval threshold, which is assigned by the modeler. While a chunk’s activation value increases each time it is retrieved, its activation value will decay over time when it is not retrieved. Depending on the type of the request from declarative memory, the chunk with the highest activation value is retrieved. The optimized learning equation which is used in the instance-based learning model to calculate the learning of base-level activation for a chunk *i* is as follows:

$$B_{i}=In(n/\left(1-d\right))-d^{*}In\left(L\right)$$

Here, n is the number of presentations of chunk i, L is the lifetime of chunk i (the time since its creation), and d is the decay parameter.

In ACT-R, *reinforcement learning* occurs when the utilities (U) that are attached to production rules are updated based on experience (Taatgen et al., 2006). A strategy (i.e., zero-order, first-order, second-order) that has the highest probability of success is used more often. Utilities can be updated based on reward (*R*). Reward can be associated with specific strategies, which are implemented by production rules. The reward is propagated back to all the previous production rules that are between the current reward and the previous reward. The reward that is propagated back is calculated with the assigned reward value minus the time passed since the execution of the related production rule, meaning that more distant production rules receive less reward. If the assigned reward is zero, the production rules related to the execution of a production rule that is associated with the reward will receive negative reward (punishment). Based on these mechanisms, a model learns to apply the best strategy for a given task. The utility learning equation which is used in the reinforcement learning model is as follows:

$$U_{i}\left(n\right)=U_{i}\left(n-1\right)+\alpha \left[R_{i}\left(n\right)-U_{i}\left(n-1\right)\right]$$

Here, U_i_ (n-1) is the utility of a production i after its n-1st application, R_i_ (n) is the reward the production receives for its *n*th application and *α* is the learning rate.

In the following two sections, we explain the details of how the instance-based learning and reinforcement learning model select ToM strategies based on experience. Subsequently, we explain the general assumptions and the reasoning steps in both models.

### How the Instance-Based Learning Model Goes Through Transitions

The assumption of the instance-based learning model is that possible strategies to apply different levels of ToM reasoning in the second-order false belief task (i.e., zero-order, first-order, second-order) are represented as chunks in declarative memory. The model uses these to select its strategy at the start of a problem: It will retrieve the strategy with the highest activation, after which production rules carry out that strategy. Based on the success, the model will either strengthen a successful strategy chunk, or will add or strengthen an alternative strategy if the current one failed. Our instance-based learning model uses the same mechanism for strategy selection as in Meijering et al.’s (2014) ACT-R model that shows adults’ strategy selection in a ToM game. The core idea of their model was that people in general use ToM strategies that are “as simple as possible, as complex as necessary” so as to deal with the high cognitive demands of a task.

The instance-based learning model starts with only a single strategy, which is stored in declarative memory as a chunk: the zero-order ToM strategy. Similar to young children’s daily life experiences, the zero-order ToM strategy chunk’s base level activation is set to a high value to represent that the model has a lot of experience in using this strategy. In line with this simplistic zero-order ToM strategy that is based on the real location of the object, the model gives the answer “TV stand” (see **Figure 1**) to the second-order false belief question (“Where does Ayla think that Murat will look for the chocolate?”). However, as this is not the correct answer to the second-order false belief question (drawer), the model gets the feedback “Wrong” without any further explanation. This stage of the model in which the zero-order ToM strategy seems to be more salient than the first-order ToM strategy represents children who are able to attribute first-order false beliefs but are lacking experience in applying the first-order ToM strategy.

Given this feedback, the model increments the reasoning strategy just used (zero-order) one level up and enters a new strategy chunk in declarative memory: a chunk that represents the first-order ToM strategy, in which the former (zero-order) strategy is now attributed to Murat. This makes it a first-order ToM strategy because this time the model gives an answer based on what the reality is (zero-order) from Murat’s perspective (first-order). Because the model has more experience with the zero-order ToM strategy, the activation of the zero-order ToM strategy chunk at first is higher than the recently added first-order ToM strategy chunk. This causes the model to retrieve the zero-order ToM strategy chunk instead of the first-order ToM strategy chunk in the next few repetitions of the task. Thus, the model still gives an answer to the second-order false belief question based on zero-order reasoning. Nevertheless, each time that the model gets the negative feedback “Wrong,” it creates a first-order ToM strategy chunk. As the identical chunks are merged in the declarative memory, the first-order ToM strategy chunk’s activation value increases.

When the activation value of the first-order ToM strategy chunk is high enough for its successful retrieval, the model gives an answer to the second-order false belief question based on first-order reasoning (toy box). Again, this is not a correct answer to the second-order false belief question (drawer). After the model gets the feedback “Wrong,” it again increments the first-order strategy by attributing a first-order ToM strategy to another agent (Ayla), which makes it a second-order ToM strategy, because this time the model gives an answer based on what Murat *thinks* (first-order) from Ayla’s perspective (second-order). This second-order strategy gives the correct answer (drawer). Given the positive feedback “Correct,” the second-order ToM strategy is further strengthened and finally becomes stable. In theory, there is no limitation on the level of strategy chunks. Nevertheless, in practice there is no need to use a very high level of reasoning (Meijering et al., 2014), and even if one tries to apply more than third-order or fourth-order ToM reasoning, it will be very hard to apply that strategy due to memory limitations (see Kinderman et al., 1998 and Stiller and Dunbar, 2007 for adults’ limitations in higher levels of ToM reasoning).

### How the Reinforcement Learning Model Goes Through Transitions

Unlike our instance-based learning model in which the reasoning strategy chunks (i.e., zero-order, first-order, second-order) are added to the declarative memory over the repetition of the task, in the reinforcement learning model the reasoning strategies are implemented with production rules. Therefore, the model selects one of these strategies based on their utilities.

Similar to the zero-order ToM strategy chunk’s relatively high base-level activation in the instance-based learning model, the utility of the production rule of the zero-order ToM strategy is arbitrarily set to a much higher value (100) than the production rules that represent the first-order (25) and second-order (5) ToM strategies. Thus, initially the reinforcement learning model gives zero-order answers. The relativity of those values is the assumption that the model has a lot of experience with the zero-order ToM strategy, and more experience with the first-order ToM strategy than the second-order ToM strategy, based on children’s development.

After the reinforcement learning model gives the zero-order answer (TV stand), it gets the feedback “Wrong.” Based on this feedback, the zero-order ToM strategy production rule gets zero reward. As explained in Section “The Relevant Mechanisms of the Cognitive Architecture ACT-R,” this mechanism decreases the utility of the zero-order ToM strategy production rule. The first-order ToM strategy production rules are executed when the utility of the zero-order ToM strategy decreases enough (to around 25). After selection of a first-order ToM strategy, the model again gets zero reward. This reward is propagated back through the other production rules of the first-order ToM strategy up to the production rule that gives the zero-order answer. Finally, when the model is able to execute the second-order strategy and to give the correct answer (drawer), it gets a higher reward (20). Therefore, the second-order ToM strategy becomes the dominant strategy. Importantly, as we discussed in the Introduction, the selection of ToM strategies is purely based on a utility mechanism, thus it is implicit compared to the explicit ToM strategy of the instance-based model.

### General Assumptions and Reasoning Steps in Both Models

Even though our models are not dependent on the particular features of a specific second-order false belief task, we modeled children’s reasoning in the prototype of a modified version of the standard second-order false belief that we explained in the introduction (see **Figure 1**). One of the assumptions of our models is that the models already heard the second-order false belief story and are ready to answer the second-order false belief question “Where does Ayla think that Murat will look for the chocolate?” Thus, the story facts are already in the models’ declarative memory. The models do not store the entire story in their declarative memory but just the facts that are related to answering the second-order false belief question. **Table 1** presents the verbal representation of those story facts^5^. As can be seen from **Table 1**, each story fact is associated with a specific time, meaning that the model knows which events happened after, before, or at the same time as a certain other event. Unlike the reinforcement learning model, the instance-based learning model starts with a zero-order ToM strategy chunk in declarative memory in addition to the story facts.

**Table 1:** The representations of story facts that are initially in declarative memory before the model starts to reason for the second-order false belief question.

“Murat put the chocolate into the drawer at time t1”	**Figure 1C**
“Ayla put the chocolate into the toy box at time t2”	**Figure 1D**
“Murat saw Ayla at time t2”	**Figure 1D**
“Ayla did not see Murat at time t2”	**Figure 1D**
“The mother put the chocolate into the TV stand at time t3”	**Figure 1E**


Both models have the following task-independent knowledge to answer the second-order false belief question (see Stenning and van Lambalgen, 2008 for an example formalization of a first-order false belief task by using similar task-independent knowledge): (i) The location of an object changes by an action toward that object; (ii) ‘Seeing leads to knowing,’ which is acquired by children around the age of 3 (Pratt and Bryant, 1990); (iii) People search for objects at the location where they have last seen them unless they are informed that there is a change in the location of the object; (iv) Other people reason ‘like me.’ For instance, based on the task-independent knowledge (ii), both models can infer that Murat *knows* that the chocolate is in the toy box once the story fact “Murat saw Ayla at time 2” (**Table 1**, row 3) has been retrieved.

**Table 2** shows the steps that have been implemented to give an answer for the second-order false belief question in the instance-based model and the reinforcement learning model. As can be seen from **Table 2**, both models always use the same set of production rules in the first two steps, which represent reasoning about reality. This feature of the models reflects the usual process of a person’s reasoning from his/her own point of view (Epley et al., 2004). Although the instance-based and reinforcement learning models have different learning mechanisms and different underlying assumptions for the selection of the reasoning strategies, the general idea for both models is that they reason about another agent as if the other model is reasoning “like me,” and use this “like me” strategy recursively. Note that we implemented the models to answer the second-order false belief question, therefore, the second-order ToM strategy becomes stable over repetition. However, when the models hear the first-order false belief question “Where will Murat look for the chocolate?”, they will use a first-order strategy instead of a second-order reasoning strategy if the activation of the first-order strategy is higher than the zero-order strategy in the instance-based learning model and if the utility of the first-order reasoning strategy is higher than that of the zero-order strategy in the reinforcement learning model.

**Table 2:** The steps that are implemented to give an answer for the second-order false belief question for the instance-based and the reinforcement learning models.

Instance-based learning model	Reinforcement learning model
(1) Retrieve a story fact that has an action verb in its slots.	(1) Retrieve a story fact that has an action verb in its slots.
(2) Check the time slot of the retrieved story fact and if it is not the latest fact, request the latest one.	(2) Check the time slot of the retrieved story fact and if it is not the latest fact, request the latest one.
(3) Request a retrieval of one of the strategy chunks from declarative memory.	(3) If the production rule that represents the zero-order strategy has the highest utility, give an answer based on the location slot of the chunk that is retrieved in the second step. If the production rule that represents k^th^-order strategy (0 < k ≤ 2) has the highest utility, apply that strategy to give an answer by reasoning as if that person employs (k-1)^th^-order reasoning.
(4) If the zero-order strategy is retrieved, give an answer based on the location slot of the chunk that is retrieved in the second step. If the k^th^-order strategy (0 < k ≤ 2) is retrieved, determine whose knowledge the question is about and give the answer by reasoning as if that person employs (k-1)^th^-order reasoning.	(4) Based on the feedback (i.e., Correct/Wrong), give the reward associated with that level of reasoning strategy.
(5) Based on the feedback (i.e., Correct/Wrong), strengthen the successful strategy chunk, or will add or strengthen an alternative strategy if the current one failed.	


In more detail, the k*^th^*-order reasoning production rules shared by both of the models are as follows: If the *zero-order strategy* is retrieved, give an answer based on the location slot of the chunk that has been retrieved previously. If the *first-order strategy* is retrieved, check whether Murat saw the object in that location or not. If Murat saw the object in that location, give an answer based on the location slot of the chunk, otherwise retrieve a chunk in which Murat saw the object previously and give an answer based on the location slot of that chunk. If the *second-order strategy* is retrieved, repeat the procedure of the first-order strategy, however, this time, instead of giving the answer from Murat’s perspective, check whether Ayla saw Murat at that time. If Ayla did not see Murat, then retrieve a chunk in which Murat put the object and give an answer based on the location slot of that chunk^6^.

Therefore, the generalized explanation of this procedure can be summarized as follows: If the *k^th^-order strategy* (0 < k ≤ 2) is retrieved, determine whose knowledge the question is about and give the answer by reasoning as if that person employs (k-1)*^th^*-order reasoning.

### Parameters

Following the criteria that we stated in Section “Previous Models of False Belief Reasoning,” we did not introduce any new parameters in addition to ACT-R’s own parameters. Moreover, all the parameters were set to their default values, except the retrieval threshold and the instantaneous noise parameters for the instance-based learning model and the utility noise parameter for the reinforcement learning model (there are no default values in ACT-R for those parameters). As previous empirical studies showed that children mostly give correct answers for the control questions (Flobbe et al., 2008; Hollebrandse et al., 2008), the retrieval threshold was set to an arbitrary low value (-5), so that the model is always able to retrieve the story facts. Thus, our models’ failure in the second-order false belief task is not due to forgetting some of the story facts but due to inappropriate strategy selection.

For the reinforcement learning model, we turned the utility learning parameter on. Similar to activations, noise is added to utilities. Noise is controlled by the utility noise parameter, which is set to 3.

### Model Results and Predictions

In this section, we present the results and predictions of the instance-based model and the reinforcement learning model.

#### Instance-Based Learning Model Results

**FIGURE 2:**
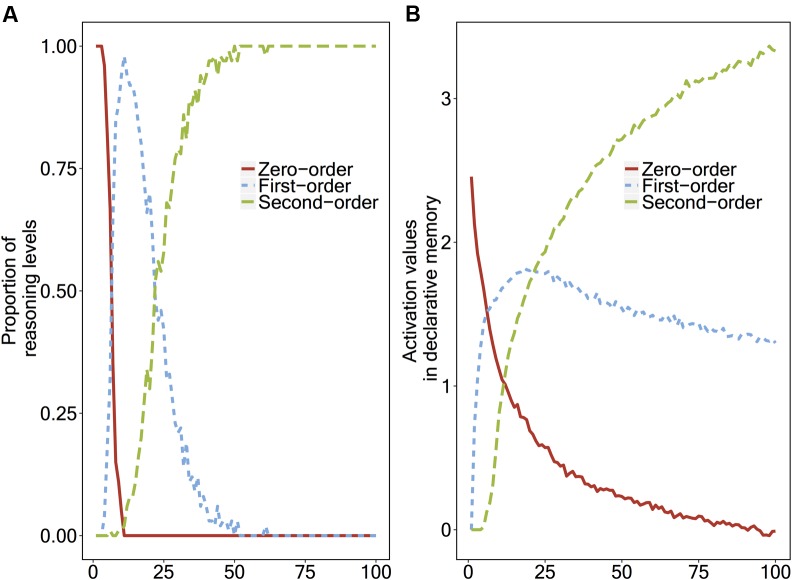
**(A)** Proportions of the reasoning level that the instance-based learning model applies, and **(B)** The activation values of the reasoning level strategy chunks, plotted as a function of number of repetitions.

To show the developmental transitions from zero-order to second-order reasoning, we ran the model 100 times per ‘virtual child,’ indicating that one child learns to apply second-order reasoning over time by gaining experience. To average the results across 100 children, we made 100 repetitions of the second-order false belief task for each ‘virtual child.’ Thus, we ran the model 10,000 times in total. For each ‘virtual child,’ the initial activation of the zero-order reasoning chunk was set to 6, indicating that children have a lot of experience with zero-order reasoning. **Figure 2A** shows the proportion of the levels of reasoning the model applies, and **Figure 2B** shows the activation values of the strategy chunks over time.

In **Figure 2A**, around the 12th repetition, the model uses the first-order strategy (60%) more than the zero-order strategy (40%). Around the 26th repetition, the model uses both second-order (50%) and first-order (50%) reasoning with an equal chance, and around the 40th repetition the model uses second-order reasoning (80%) much more than first-order reasoning (20%). Finally, around the 50th repetition, the second-order reasoning strategy becomes stable (100%).

As we explained in Section “How the Instance-Based Learning Model Goes Through Transitions,” the transitions in the strategy chunks are based on the activation of those chunks. In **Figure 2B**, around the 10th repetition, the first-order ToM strategy chunk’s activation becomes higher than that of the zero-order ToM strategy chunk which leads the model to apply first-order ToM instead of the zero-order ToM. Finally, around the 26th repetition, the activation of the second-order ToM strategy chunk’s activation becomes higher than that of the other strategies, so that the model makes a second-order belief attribution to Ayla.

#### Reinforcement Learning Model Results

**FIGURE 3:**
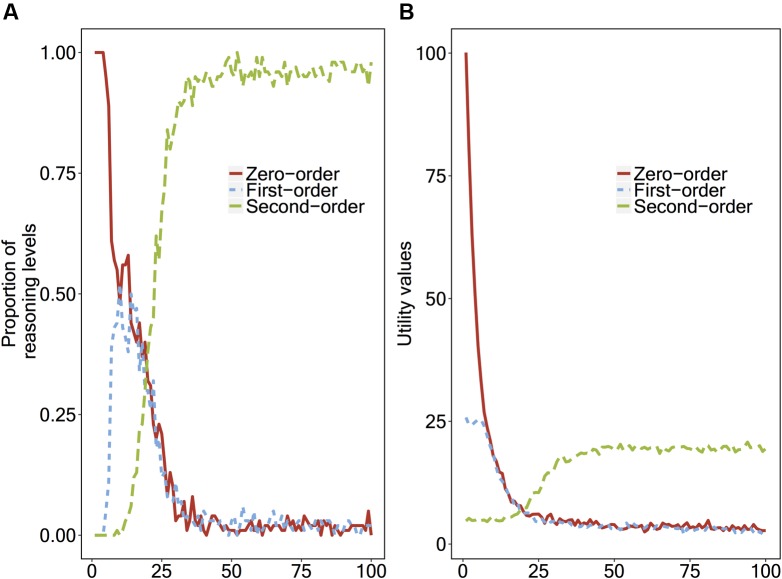
**(A)** Proportions of the reasoning level that the reinforcement learning model applies, and **(B)** The utility values of the strategies, plotted as a function of number of repetitions.

Similar to the instance-based learning model, we ran the reinforcement learning model 10,000 times in total to average the results across 100 ‘virtual children’ repeating the second-order false belief task 100 times each. **Figure 3A** shows the proportion of the levels of reasoning that the reinforcement learning model applies, and **Figure 3B** shows the utility values of the strategies.

Different from the instance-based learning model’s results (see **Figure 3A**), the reinforcement learning model does not go through the transitions in a stepwise fashion. Until around the 10th repetition, the model uses a zero-order strategy and a first-order strategy randomly (50%/50%), and does not use the second-order strategy. Before the model starts to use the second-order strategy more often (60%) than the other two strategies (around 30th repetition), it uses both the zero-order and first-order strategies, and not necessarily the first-order strategy more often than the zero-order one. Finally, around the 50th repetition, the second-order reasoning strategy becomes stable (100%).

#### Comparing the Predictions of the Two Models

(1) The first predictions of the instance-based and reinforcement learning models are related to children’s errors in second-order false belief tasks. Following the pattern in **Figure 2A**, the *instance-based learning* model predicts that children who do not have enough experience with second-order reasoning give first-order answers to the second-order false belief question. On the other hand, following the pattern in **Figure 3A**, once the *reinforcement learning* model is able to execute the first-order ToM strategy, it selects between zero-order and first-order ToM strategies randomly, on the basis of noise. Thus, the reinforcement learning model does not predict that children’s wrong answers would most of the time be based on the first-order reasoning strategy^7^.
(2) The second predictions of both models are related to learning second-order false belief reasoning over time based on the given feedback. Both of the models predict that children who have enough experience with first-order ToM reasoning but not with *second-order* ToM reasoning can learn to apply second-order ToM without any need to have further explanations of why their answer is wrong. This prediction contrasts with previous findings showing that 4-year-old children’s performance on *first-order* false belief tasks cannot be improved when they are trained on false belief tasks with feedback without giving detailed explanations (Clements et al., 2000).
(3) Although both models predict that training children with feedback “Wrong” is sufficient to accelerate their development of second-order false belief reasoning, the *instance-based* learning model provides an additional underlying prediction. Because the instance-based learning model explicitly increments its wrong first-order ToM strategy to the correct second-order ToM strategy, if the model would receive feedback *together with further explanations* (not only “Wrong”), the odds of selecting the correct strategy would increase. In contrast, providing feedback *with further explanations* does not provide any useful additional information for the reinforcement learning model.

## Experimental Validation of the Instance-Based Learning Model

In this section, we present the experimental validation of our instance-based learning model’s first prediction, which proposes that 5-year-old children will give first-order ToM answers in the second-order false belief task.

### Participants

In order to test our model-based predictions related to children’s wrong answers, we analyzed the cross-sectional data (pre-test) of a larger training study that includes a sample of 79 Dutch 5- to 6-year-old children (38 female, *M*_age_ = 5.7 years, *SE* = 0.04, range: 5.0 – 6.8 years). All children were recruited from a primary school in Groningen, the Netherlands from predominantly upper-middle-class families. The children were tested individually in their school in a separate room.

Approval and parental consent was obtained in accordance with Dutch law. Because we are interested in children’s wrong answers, seven children who gave correct answers for both of the second-order false belief questions were excluded from our analysis. Therefore, the analysis included the results of 72 children (36 female, *M*_age_ = 5.7 years, *SE* = 0.05, range: 5.0 – 6.8).^8^

### Materials

Children’s answers to 17 different second-order false belief stories of two different types^9^ were analyzed: (i) 3 ‘Three locations’ stories, (ii) 14 ‘Three goals’ stories. Within the story types, we always kept the structure the same while we changed the name, gender and appearance of the protagonists, along with the objects and the locations, or goals. Stories of both types were constructed in such a way that it is possible to infer whether children’s possible answers to second-order false belief questions correspond to zero-order, first-order, or second-order reasoning. Control questions including the reality (zero-order) and first-order false belief questions were asked before the second-order false belief questions, to test that children did not have major memory problems about the story facts, linguistic problems about the questions, and first-order false belief attribution.

‘Three locations’ stories were constructed based on Flobbe et al.’s (2008) ‘Chocolate Bar’ story (see **Figure 1**). As we discussed in the introduction, inspired by Hollebrandse et al.’s (2008), we modified Flobbe et al.’s (2008) Chocolate Bar story in such a way that it is possible to distinguish children’s possible reasoning levels (i.e. zero-order, first-order, second-order) from their answers to second-order false belief questions. Before the second-order false belief question (e.g., “Where does Ayla think that Murat will look for the chocolate?”) and the justification question (“Why?”), we asked four control questions. The first and second control questions were asked after **Figure 1D** as follows: (i) “Does Murat know that Ayla put the chocolate into the toy box?”, (ii) “Does Ayla know that Murat saw her putting the chocolate into the toy box?”. The third control question (zero-order ToM) was asked after the fifth episode in **Figure 1E**: (iii) “Where is the chocolate now?”. Subsequently, the fourth control question (first-order false belief question) was asked: (iv) “Where will Murat look for the chocolate?”.

‘Three goals’ stories included and extended the stories used in Hollebrandse et al.’s (2008) study. One of the examples of this story type is as follows: “Ruben and Myrthe play in their room. Myrthe tells Ruben that she will go to buy chocolate-chip cookies from the bake sale at the church and she leaves the house. After that, their mother comes home and tells Ruben that she just visited the bake sale. Ruben asks his mother whether they have chocolate-chip cookies at the bake sale. The mother says, ‘No, they have only apple pies.’ Then Ruben says, ‘Oh, then Myrthe will buy an apple pie’.” At this point, the experimenter asked the first control question: “Does Myrthe know that they sell only apple pies in the market?”. The story continued: “Meanwhile, Myrthe is at the bake sale and asks for the chocolate-chip cookies. The saleswoman says, ‘Sorry, we only have muffins.’ Myrthe buys some muffins and goes back home.” Now, the second control question “Does Ruben know that Myrthe bought muffins?” and the first-order false belief question “What does Ruben think they sell in the market?” together with the justification question “Why does he think that?” were asked. Then the story proceeded: “While she is on her way home, she meets the mailman and tells him that she bought some muffins for her brother Ruben. The mailman asks her what Ruben thinks that she bought.” Then, the experimenter asked the participant the second-order false belief question: “What was Myrthe’s answer to the mailman?”. The justification question “Why?” was asked after the second-order false belief question.

There are three possible answers to the second-order false belief question that children might report: chocolate-chip cookies, which Myrthe told Ruben initially (correct second-order answer); an apple pie, which the mother told Ruben (first-order answer); and muffins, which Myrthe really bought (zero-order answer).

### Procedure

All the stories were presented to the children on a 15-inch MacBook Pro and were implemented with Psychopy2 v.1.78.01. All the sessions were recorded with QuickTime. If a child gave a correct answer for a second-order false belief question, his or her score was coded as 1, while incorrect answers were coded as “zero-order” or “first-order” or “I don’t know,” based on the given answer.

The two different types of second-order false belief stories were pseudo-randomly drawn from a pool that contained 17 different false belief stories (3 ‘Three location’ stories and 14 ‘Three goals’ stories). Drawings illustrating the story episodes were presented one by one, together with the corresponding audio recordings. The drawings remained visible throughout the story. A child was never tested on the same story twice. Children did not get any feedback.

### Results

**FIGURE 4:**
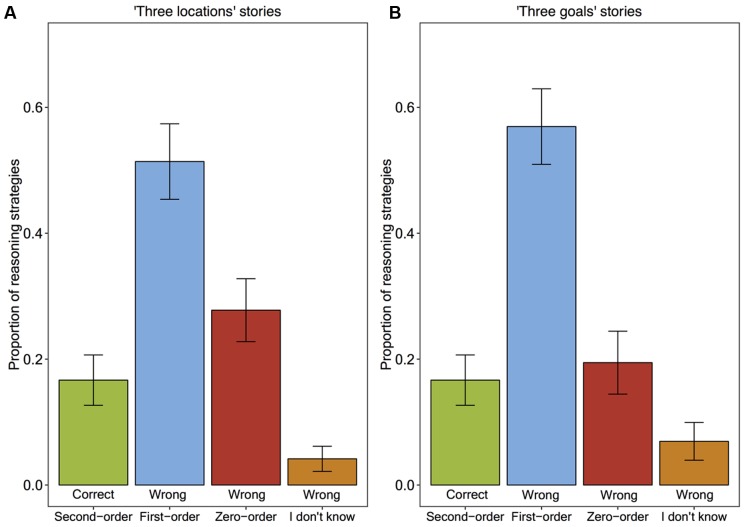
**The proportion of children’s level of ToM reasoning strategies when answering the second-order false belief questions** **(A)** in ‘Three locations’ stories, **(B)** in ‘Three goals’ stories.

**Figure 4** shows the proportion of children’s level of ToM reasoning for the second-order false belief questions. Confirming our instance-based learning model’s prediction, most of the time children’s wrong answers to the second-order false belief questions were first-order ToM answers (51% in the ‘Three locations’ stories, and 57% in the ‘Three goals’ stories) and relatively few of the answers were zero-order ToM answers (28% in the ‘Three locations’ stories, and 19% in the ‘Three goals’ stories). Overall, 17% of the second-order false belief answers were correct and 83% of them were wrong. Whereas 65% of the wrong answers were based on a first-order ToM strategy, 29% of them were based on a zero-order strategy, and the remaining 6% was “I don’t know.”

A chi-square test of independence was performed to examine the relation between the two story types and the children’s levels of reasoning in their wrong answers. The relation between these variables was not significant. For this reason, we merged the data over the story types and conducted a chi-square test of goodness-of-fit to determine whether the zero-order, first-order and “I don’t know” answers were given equally often. Different levels of children’s wrong answers were not equal in the population, *X^2^*(2, *N* = 119) = 64.76, *p* < 0.001.

**Table 3** shows the percentages of correct answers for each type of question (i.e., control, first-order false belief, second-order false belief). As can be seen from **Table 3**, children almost all the time gave correct answers for the control questions for both of the story types. Their percentage of correct answers for the first-order false belief questions was lower in ‘Three locations’ stories (81%) than ‘Three goals’ stories (93%)^10^. Children’s correct answers to the second-order false belief questions were lower than the chance level 33% for both for the ‘Three locations’ stories (17%) and the ‘Three goals’ stories (17%).

**Table 3:** The percentages of correct answers and standard errors (in parenthesis) for the control, first-order false belief and second-order false belief questions for both ‘Three locations’ and ‘Three goals’ story types.

Questions	‘Three locations’	‘Three goals’
Control	95% (0.02)	96% (0.01)
First-order false belief	81% (0.05)	93% (0.03)
Second-order false belief	17% (0.05)	17% (0.05)


## Discussion, Conclusion and Future Work

In order to provide a procedural account for children’s strategy selection while they are answering second-order false belief questions, we constructed two computational cognitive models: an instance-based model and a reinforcement learning model. Importantly, we did not introduce any additional parameters to the core cognitive architecture ACT-R to trigger a transition from incorrect to correct answers and we stated a model-based prediction before conducting our empirical study. Our main finding in this study is the confirmation of our instance-based learning model’s prediction that 5- to 6-year-old children who have enough experience in first-order ToM but fail in second-order false belief tasks apply a first-order ToM strategy in the second-order false belief tasks. Our empirical results showed that most of the wrong answers to the second-order false belief questions were based on a first-order ToM strategy (65%) and few of the wrong answers were based on a zero-order strategy (29%). Note that, as we presented in Section “Comparing the Predictions of the Two Models”, the reinforcement learning model did not predict that children’s wrong answers would most of the time be based on the first-order reasoning strategy. Before highlighting the differences between the two models and explaining their meaning in children’s second-order ToM development, we would like to discuss our empirical findings compared to the previous findings in the second-order ToM literature.

Our empirical findings are consistent with Hollebrandse et al.’s (2008) results that we explained in the Introduction. Considering that children were older in Hollebrandse et al.’s (2008) study, the higher proportion of second-order ToM answers (58%) and the lack of zero-order ToM answers in their experimental results compared to ours are in line with our instance-based model’s first prediction that children who have enough experience with first-order false belief reasoning but not with second-order reasoning do not give zero-order answers but first-order answers for the second-order false belief question. On the other hand, our empirical findings are in contrast with de Villiers et al.’s (2014) preliminary results showing that most 5- to 6-year-olds in low-income preschools gave zero-order ToM answers (60%) in the second-order false belief task, and fewer of the answers were based on the first-order ToM strategy (20%). We argue that the difference between our results and de Villiers et al.’s (2014) preliminary results can be attributed to their sample’s low-income socioeconomic status, compared to our sample’s upper-middle income socioeconomic status (see Holmes et al., 1996; Cole and Mitchell, 2000 for significant correlations between family socioeconomic status and individual differences in false belief performance). Moreover, the school at which we tested the children is called ‘Excellence school,’ meaning that children’s scores on the national tests are almost at the upper limit. The school’s success comes from their adaptive education, which tries to ensure that both the gifted and the weaker students perform at their individual maximum. These educational and socioeconomic differences might be a possible explanation for the different results.

Our empirical findings confirm the instance-based learning model’s prediction about children’s wrong answers. One could argue that children may have been primed to give first-order answers because the first-order false belief questions were always asked right before the second-order false belief questions. This interpretation suggests that children only retrieved the most recent strategy chunk (i.e., first-order) or alternatively that they retrieved the most active location and gave their answers accordingly. However, even if the zero-order ToM question (“Where is the chocolate?”) were asked in between the first-order and the second-order false belief questions, we believe that children would usually give first-order answers. This is because 4-year-old children can already pass the first-order false belief task and as long as 5-year-old children have enough experience with first-order ToM reasoning, they would usually give first-order answers instead of zero-order answers. de Villiers et al.’s (2014) findings can be seen as evidence that asking first-order false belief questions right before the second-order false belief questions does not necessarily prime children to give first-order answers.

Moreover, the linguistic literature that shows that children respond to the embedded part of the second-order false belief question (i.e., first-order ToM reasoning) can be used as evidence that children do not only repeat the last given answer but that they have problems with selecting a strategy or with processing embedded structures (Astington et al., 2002; de Villiers et al., 2014).

In addition to the empirical validation of our instance-based learning model’s prediction, our modeling approach allows us to provide insight about children’s development of second-order false belief reasoning. Unlike our reinforcement learning model, our instance-based learning model selects a ToM strategy which is “as simple as possible, as complex as necessary” (Meijering et al., 2014). Similar to this approach, Goodman et al. (2006) used a Bayesian Occam’s razor effect to explain that children initially reason from their own perspective (zero-order ToM) in first-order false belief tasks and then with accumulated evidence revise their strategy to taking into account another agent’s perspective (first-order ToM). The main difference between Goodman et al. (2006) and our modeling approach can be formulated in terms of Marr’s (1982) levels of analysis. While their model takes place at the computational level only, our models also reflect the algorithmic level.

Using the “as simple as possible, as complex as necessary” approach is also in line with the previous literature on adults’ ToM reasoning in strategic games in which adults are found to start applying lower levels of ToM strategies and slowly increment their level of ToM strategy when it is necessary (Hedden and Zhang, 2002; Camerer et al., 2004; Wright and Leyton-Brown, 2010; Goodie et al., 2012) and a cost-benefit approach in which a strategy with the lowest cognitive effort (cost) and the best accuracy (benefit) is selected (Payne et al., 1993; Rieskamp and Otto, 2006). Analogous to those approaches, our instance-based learning model first applies the most salient and least cognitively effortful strategy, and then if that strategy does not work increments the strategy one level higher, instead of two levels higher or more. Unlike the reinforcement learning model, this approach proposes that children’s strategy revision is explicit.

One of the assumptions of our models was that initially children have a lot of experience with the zero-order ToM strategy because they perceive the world from their own perspective. On the other hand, once children have enough experience with first-order ToM reasoning they use the first-order ToM strategy in second-order false belief tasks. To validate the assumption about children’s experiences with the different orders of ToM strategies, further research is needed in which children’s everyday life experiences are investigated.

Although the reinforcement learning model and the instance-based learning model provided different predictions about children’s systematic errors in second-order false belief tasks, both models predicted that with exposure to second-order ToM reasoning, 5-year-olds can learn to apply correct second-order ToM strategy with feedback “Wrong” without any need for further explanations of why their answer was wrong. This prediction contrasts with the first-order ToM literature showing that 4-year-olds’ first-order false belief reasoning cannot be improved without further explanations (Clements et al., 2000). On the other hand, different from the reinforcement learning model, the instance-based learning model predicts that children who get feedback with explanations are more likely to revise their wrong first-order ToM strategy to the correct second-order ToM strategy. To test these model-based predictions, we are currently conducting a training study in which children hear different second-order false belief stories in two different training days with four different experimental conditions (i.e., feedback without explanation, feedback with explanation, no feedback at all, and a control condition in which children are trained with neutral stories that do not involve ToM reasoning). Confirming our instance-based learning model’s predictions, our preliminary results show that children’s performance from pre-test to post-test significantly increases in the feedback without explanation condition and that children who receive feedback with further explanations improve more than children who receive feedback without any further explanations (Arslan et al., 2015a). The preliminary results of our training study signal that 5-year-olds’ failure in second-order false belief tasks cannot be due to maturation, related to increasing functionality of mechanisms of the brain as in Hiatt and Trafton (2015) unless there is a stimulus-triggered brain maturation.

Because our model starts to reason from its own perspective (zero-order ToM), and then takes into account another agent’s beliefs (first-order ToM), and finally uses ToM recursively (second-order ToM), we can predict that children will look first to the picture that represents reality (**Figure 1E**), then to the picture that represents the first-order ToM strategy (**Figure 1D**), and finally to the picture that represents the second-order ToM strategy (**Figure 1C**). An eye-tracking study in which we can analyze children’s eye movements when children are answering the second-order false belief questions can provide more insight about the underlying processes.

Does selecting the correct reasoning strategy mean that children can perfectly apply that strategy? As we explained in Section “How the Reinforcement Learning Model Goes Through Transitions,” once our instance-based learning model retrieves a second-order ToM strategy, it always gives the correct answer. More specifically, we have ready production rules in the model, which apply second-order ToM reasoning perfectly, when the second-order strategy has been selected. In line with the *complexity* explanation, we believe that selecting the correct strategy is not the whole story in children’s development of second-order false belief reasoning. When children select the correct second-order ToM strategy, they might still make mistakes for different reasons, such as lack of efficiency in applying reasoning rules and internal or external distraction. We believe that our experimental results, which show that 29% of the wrong answers were still based on a zero-order strategy, as opposed to the 0% predicted by our instance-based learning model (but see Supplementary Image 1 showing that changing the noise value causes variation in the predicted percentages of wrong answers), is related to a working memory bottleneck or to distraction. The *serial processing bottleneck* (Verbrugge, 2009), which causes a lack of efficiency when children have to serially process embedded beliefs, might cause an inefficiency to use a second-order ToM strategy. This hypothesis suggests that children should serially process nested beliefs in a sequential manner. However, children who cannot pass second-order false belief tasks might have a lack of efficiency in serially processing embedded beliefs (for further evidence supporting the *serial processing bottleneck* in different cognitive domains, see Diamond et al., 2002; Hendriks et al., 2007; Van Rij et al., 2010; Ling et al., 2016) because working memory acts as a bottleneck (Borst et al., 2010), meaning that people can only hold one chunk of information in working memory at a time. More specifically, when children try to answer second-order false belief questions, e.g., “Where does Mary think that John will look for the chocolate?”, after inhibiting their own perspective, they might be holding in mind the answer of the embedded part of the question (first-order ToM reasoning), which is “Where will John look for the chocolate?”. To be able to reason about Mary’s false belief about John’s belief, children need to use efficient rules to overcome *the serial processing bottleneck*.

How do children learn ToM reasoning strategies? We surmise two possible answers to this question. The first possible answer is related to learning common sense knowledge to reason about the false belief task. Although our model learns to pass second-order false belief tasks by repeating the task itself, in real life children do not learn second-order ToM with false belief stories. Heyes and Frith (2014) propose that explicit ToM is culturally inherited, and that parental stories and “causal-explanatory” statements might be some of the possible sources of this common sense knowledge. The second possible answer is related to learning those strategies from other cognitive tasks that are not specifically related to ToM. It is unlikely that people have complex and specialized rules in their minds to give a specific answer to false belief questions, as we have in our model. Based on Arslan et al.’s (2015b) computational cognitive modeling study which showed that working memory and cognitive control strategies can contribute to children’s transitions from failure to success in first-order false belief tasks, we propose that one of the important sources of combining those complex and specialized production rules might be children’s experience in working memory strategies that they apply in their daily lives, such as counting and comparing the numbers of objects^11^. This explanation needs to be tested by designing a training study in which children are trained with simple and complex working memory tasks and their performance in second-order false belief reasoning from pre-test to post-test is assessed.

To sum up, unlike the reinforcement learning model, our instance-based model is able to predict 5-year-olds’ systematic errors in second-order false belief tasks, namely by first-order ToM strategy. Our modeling approach provides a plausible explanation for children’s systematic errors in second-order false belief tasks and shows how they revise their ToM strategy. Based on our instance-based learning model, we can surmise that 5-year-old children’s failure is due to lack of experience in using a second-order ToM strategy and that children can explicitly revise their wrong first-order ToM strategy to a correct second-order ToM strategy by exposure to second-order ToM reasoning.

## Author Contributions

BA wrote to the code of the models; and analyzed the data. BA, NT, and RV substantially contributed to the design of the model and the experiment; the interpretation of the model and the empirical results; drafting and the final approval of the version of the manuscript to be published. The agreement is accountable for all aspects of the work in ensuring that questions related to the accuracy or integrity of any part of the work are appropriately investigated and resolved.

## Conflict of Interest Statement

The authors declare that the research was conducted in the absence of any commercial or financial relationships that could be construed as a potential conflict of interest.

## Footnotes

1 We will use the general term ‘model’ to refer to the term ‘computational cognitive model’ for the rest of this study.

2 It is also called ‘egocentrism’ (Piaget, 1930) or the ‘curse of knowledge’ (Birch and Bloom, 2004). Although these different terms correspond to some underlying theoretical differences (see Mitchell et al., 1996 and Birch and Bloom, 2004 for these differences), we use the term ‘reality bias’ in this study to refer to children’s systematic errors based on reality.

3 For another type of second-order false belief question see the ‘Three goals’ story prototype in Section “Materials.”

4 See https://www.youtube.com/watch?v=u03VidFILmg for a video of young children’s failure in hide and seek.

5 For the actual representations in ACT-R, see the Supplementary Data sheet 1 or https://figshare.com/s/7c3146ad85b3e7a57cd4

6 It works similarly for the other possible first-order and second-order ToM questions in which Ayla and Murat appear (i.e., “Where will Ayla look for the chocolate?” and “Where does Murat think that Ayla will look for the chocolate?”).

7 Note that one can argue that the predictions of the reinforcement learning model may be changed by just adjusting the initial utilities of the strategies or the added noise parameters. While changing the initial utility values or the noise values would change the exact curves in **Figure 3A**, both manipulations would not change the reinforcement learning model’s prediction unless the second-order ToM strategy has higher utility than the zero-order and first-order ToM strategies, which is theoretically not plausible (see Supplementary Image 1 for examples of the models’ results with different utility values and different noise values).

8 Note that in our larger training study, children were also tested with a ToM game and a counting span task. In the ToM game, children were expected to reason about the computer’s decision and about the computer’s belief about their own decision. However, the task was too hard for our sample. For the counting span task, we constructed series of logistic regression models in order to test the effect of the counting span task score on children’s success and failure of second-order false belief questions, and also to test its effects on the different orders of children’s wrong answers (i.e., zero-order, first-order) for both of the second-order false belief questions. None of those effects were significant. For this reason, we do not present the task and its results here.

9 Children were also tested with another type of stories that are constructed based on Sullivan et al.’s (1994) Birthday Puppy story. Unlike the ‘Chocolate Bar’ story that we explained in the Introduction, this type of stories includes only two possible answers for the second-order false belief question. Therefore, it is not possible to distinguish whether children’s wrong answers are zero-order or first-order answers. For this reason, we do not include the results of this story type here. All the tasks used in this study and the different story types were presented in random order.

10 The difference between the two story types were at the significance level for children’s correct answers to first-order false belief questions, *X^2^*(1, *N* = 144) = 3.88, *p* = 0.05.

10 See (Taatgen, 2013) about the PRIMs theory for the details of how complex production rules are combined together and how experience in one domain transfers to another domain.
