# Supplementary material for: Five-Year-Olds’ Systematic Errors in Second-Order False Belief Tasks Are Due to First-Order Theory of Mind Strategy Selection: A Computational Modeling Study
Source: Front Psychol. 2017 Feb 28;8:275. doi: 10.3389/fpsyg.2017.00275 (PMC5329038; doi:10.3389/fpsyg.2017.00275)

Reinforcement Learning Model Results with Different Utility Values

In order to show that the initial utility values do not change the qualitative predictions of the model, the utility values are set to 50, 40, 20 for the zero-order, first-order and second-order ToM strategies respectively. Rest of the parameters and the number of repetitions are kept the same with the original model.

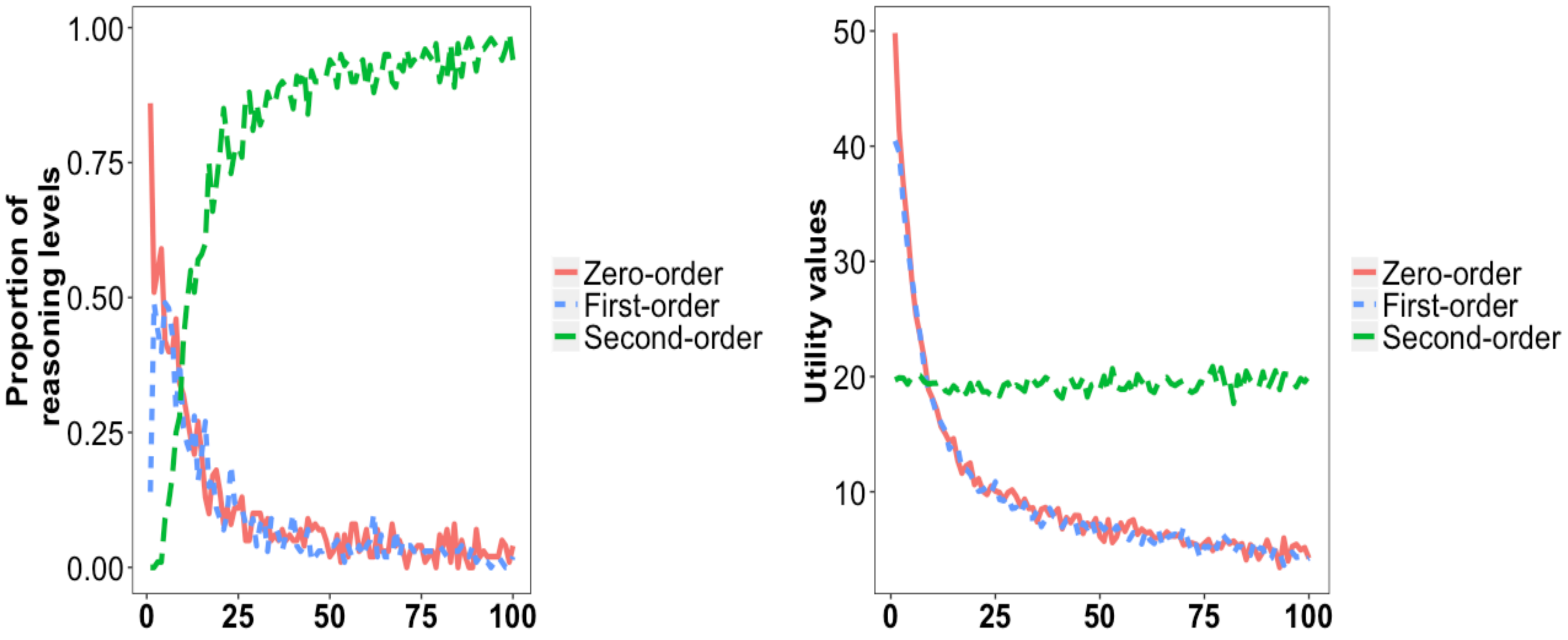

Reinforcement Learning Model Results with a Lower Noise Value

In order to show that the noise value do not change the qualitative predictions of the model, the noise value set to 1 (it is set to 3 in the original model). Rest of the parameters and the number of repetitions are kept the same with the original model.

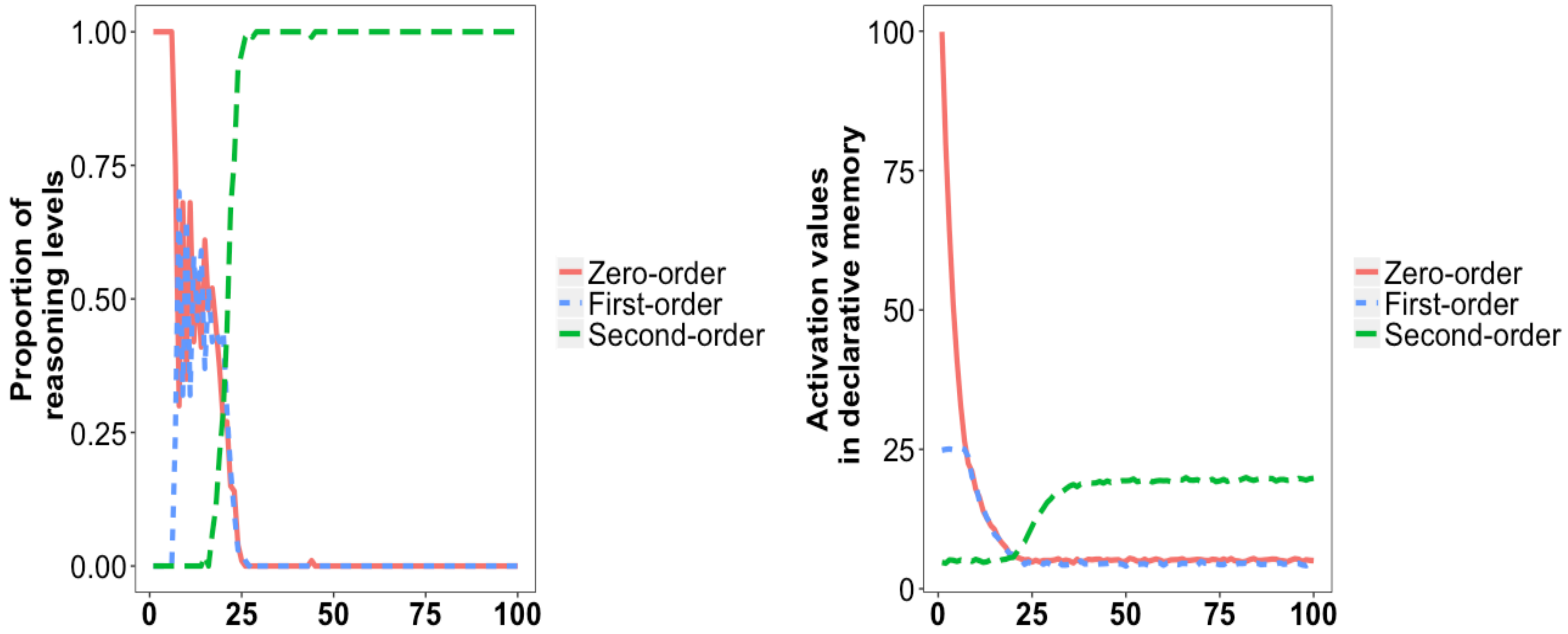

Instance-based Learning Model Results with a Higher Noise Value

In order to show that the noise value do not change the qualitative predictions of the model, the noise value set to 0.5 (it is set to 0.1 in the original model). Rest of the parameters and the number of repetitions are kept the same with the original model.

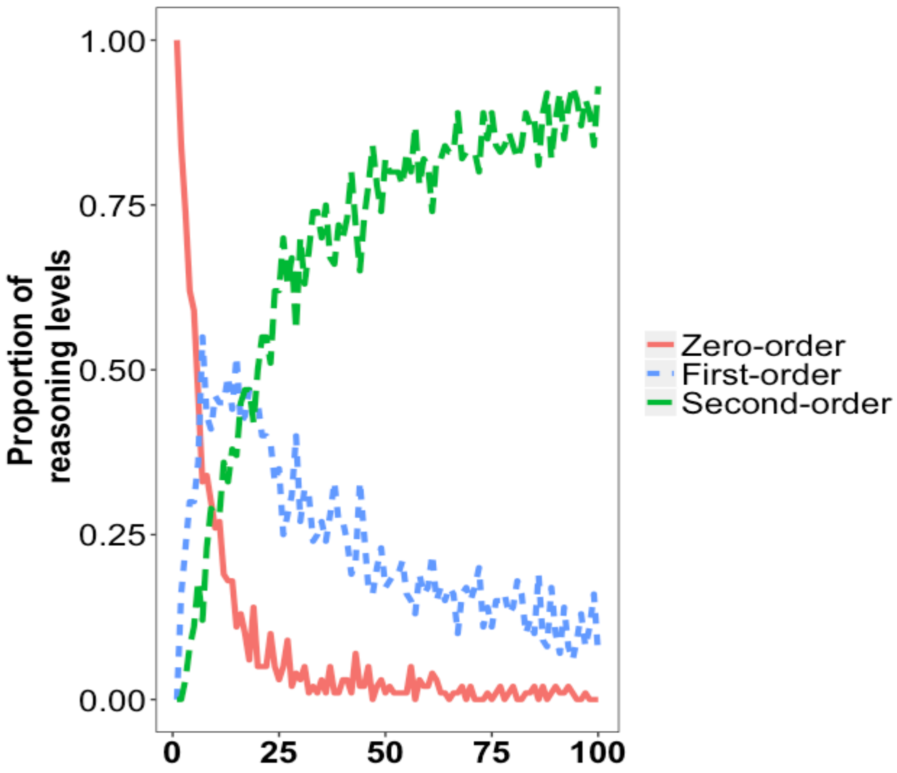

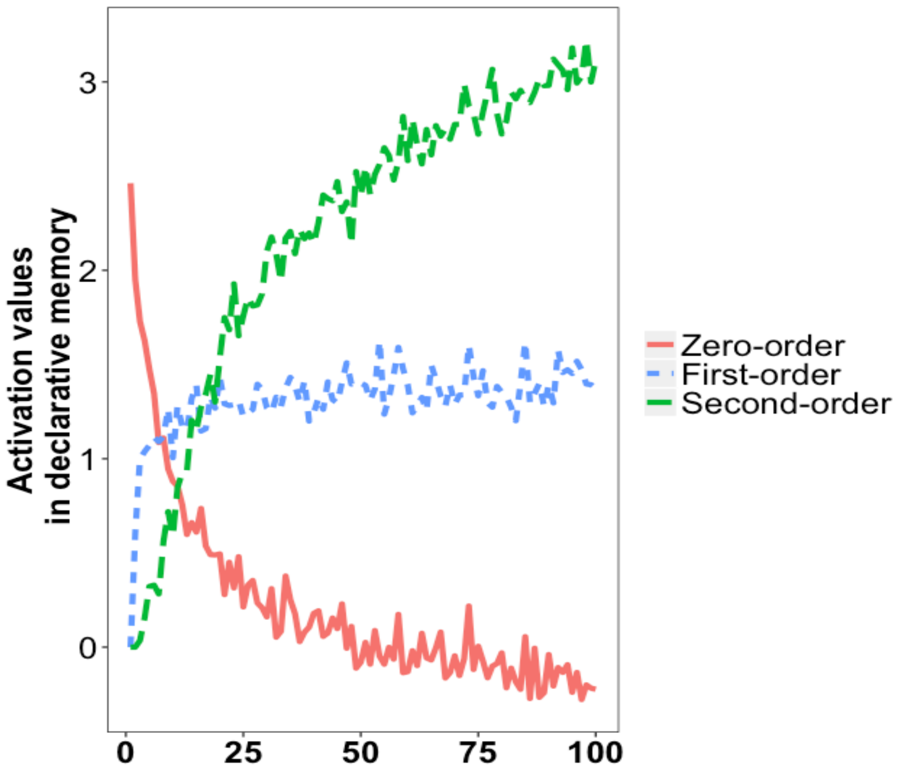

Supplement: Supplementary file 2 [file Image_1.PDF]
